# ARID2-related disorder: further delineation of the clinical phenotype of 27 novel individuals and description of an epigenetic signature

**DOI:** 10.1038/s41431-025-01798-w

**Published:** 2025-03-05

**Authors:** Clara Houdayer, Kathleen Rooney, Liselot van der Laan, Céline Bris, Mariëlle Alders, Angela Bahr, Giulia Barcia, Clarisse Battault, Anais Begemann, Dominique Bonneau, Antoine Bonnevalle, Aicha Boughalem, Alice Bourges, Marie Bournez, Ange-Line Bruel, Daniela Buhas, Floriane Carallis, Benjamin Cogné, Valérie Cormier-Daire, Julian Delanne, Tanguy Demaret, Anne-Sophie Denommé-Pichon, Julie Désir, Christèle Dubourg, Mélanie Fradin, David Geneviève, Himanshu Goel, Alice Goldenberg, Karen W. Gripp, Agnès Guichet, Anne Guimier, Adeline Jacquinet, Boris Keren, Louis Legoff, Michael A. Levy, Haley McConkey, Bryce A. Mendelsohn, Cyril Mignot, Vincent Milon, Mathilde Nizon, Beatrice Oneda, Laurent Pasquier, Olivier Patat, Christophe Philippe, Vincent Procaccio, Rebecca Procopio, Clément Prouteau, Thomas Rambaud, Anita Rauch, Raissa Relator, Sophie Rondeau, Gijs W E. Santen, Jennifer Schleit, Arthur Sorlin, Katharina Steindl, Matt Tedder, Marine Tessarech, Frédéric Tran Mau-Them, Detlef Trost, Pleuntje J Van der Sluijs, Marie Vincent, Sandra Whalen, Christel Thauvin-Robinet, Bertrand Isidor, Bekim Sadikovic, Antonio Vitobello, Estelle Colin

**Affiliations:** 1 Service de Génétique Médicale, CHU d’Angers, Angers, France; 2 Univ Angers, [CHU Angers], INSERM, CNRS, MITOVASC, SFR ICAT, F-49000 Angers, France; 3 Verspeeten Clinical Genome Centre, London Health Science Centre, London, ON Canada; 4 Department of Pathology and Laboratory Medicine, Western University, London, ON Canada; 5 Department of Human Genetics, Amsterdam Reproduction & Development Research Institute, Amsterdam University Medical Centers, University of Amsterdam, Amsterdam, The Netherlands; 6 Institute of Medical Genetics, University of Zurich, 8952 Schlieren, Switzerland; 7 Université Paris Cité, Service de Médecine Génomique des Maladies Rares, INSERM UMR 1163, Institut Imagine, Hôpital Necker - Enfants Malades, Assistance Publique-Hôpitaux de Paris, Paris, France; 8 Normandy University, UNIROUEN, INSERM U1245 and University Hospital of Rouen, Department of Genetics and Reference Centre for Developmental Disorders, F 76000, Normandy Centre for Genomic and Personalized Medicine, Rouen, France; 9 Laboratoire Cerba, Saint-Ouen-l’Aumone, France; 10 Centre de Référence Anomalies Du Développement et Syndromes Malformatifs, FHU TRANSLAD, CHU Dijon, 21000 Dijon, France; 11 Center of Genetics and Reference Centre for Intellectual Disabilities, Dijon Bourgogne University Hospital, Dijon, France; 12 Division of Medical Genetics, Department of Specialized Medicine, McGill University Health Center, Montreal, QC Canada; 13 Department of Human Genetics, McGill University, Montreal, QC Canada; 14 Laboratoire Multisites SeqOIA, Paris, France; 15 Nantes Université, CHU de Nantes, CNRS, INSERM, l’institut du thorax, F-44000 Nantes, France; 16 Nantes Université, CHU de Nantes, Service de Génétique médicale, F-44000 Nantes, France; 17 Centre de Génétique Humaine, Institut de Pathologie et Génétique, Gosselies, Belgium; 18 Service de Génétique Médicale, Centre Labellisé Anomalies du Développement de l’Ouest, CHU de Rennes, Rennes, France; 19 Montpellier University, Inserm, U1183 Montpellier, France; 20 Inserm UMR1231 GAD, Génétique des Anomalies du Développement, Université de Bourgogne, Dijon, France; 21 Hunter Genetics, Waratah, NSW Australia; 22 Division of Medical Genetics, Nemours/A.I. DuPont Hospital for Children, Wilmington, DE USA; 23 Department of Genetics, Sart Tilman University Hospital, Liège, Belgium; 24 UF de Génétique Clinique et Centre de Référence Maladies Rares des Anomalies du Développement et Syndromes Malformatifs, ERN ITHACA, APHP.Sorbonne Université, Hôpital Armand Trousseau, Paris, France; 25 Department of Medical Genetics, Kaiser Oakland Medical Center, Oakland, CA USA; 26 APHP Sorbonne Université, Département de Génétique, Centre de Référence Déficiences Intellectuelles de Causes Rares, Paris, France; 27 Department of Genetics, University Hospital of Toulouse, Toulouse, France; 28 Department of Clinical Genetics, Leiden University Medical Center, Leiden, The Netherlands; 29 Blueprint Genetics, Quest Diagnostics Company, 2505 3rd Ave, Suite 204, Seattle, WA, 98121 USA; 30 Greenwood Genetic Center, Greenwood, IN, USA

**Keywords:** Neurodevelopmental disorders, Epigenetics

## Abstract

Rare genetic variants in *ARID2* are responsible for a recently described neurodevelopmental condition called ARID2-related disorder (ARID2-RD). ARID2 belongs to PBAF, a unit of the SWI/SNF complex, which is a chromatin remodeling complex. This work aims to further delineate the phenotypic spectrum of ARID2-RD, providing clinicians with additional data for better care and aid in the future diagnosis of this condition. We obtained the genotypes and phenotypes of 27 previously unreported individuals with ARID2-RD and compared this series with findings in the literature. We also assessed peripheral blood DNA methylation profiles in individuals with ARID2-RD compared to episignatures of controls, unresolved cases, and other neurodevelopmental disorders. The main clinical features of ARID2-RD are developmental delay, speech disorders, intellectual disability (ID), behavior problems, short stature, and various dysmorphic and ectodermal features. Genome-wide differential methylation analysis revealed a global hypermethylated profile in ARID2-RD that could aid in reclassifying variants of uncertain significance. Our study doubles the number of reported individuals with *ARID2* pathogenic variants to 53. It confirms loss-of-function as a pathomechanism and shows the absence of a clear genotype-phenotype correlation. We provide evidence for a unique DNA methylation episignature for ARID2-RD and further delineate the ARID2-associated phenotype.

## Introduction

Gene expression is critically regulated by the accessibility of the DNA by the transcription machinery. It depends on the level of chromatin compaction, which can be either euchromatin or heterochromatin, and on nucleosome positioning, composition, and modification. Chromatin remodeling is a dynamic process involving two major ATP-dependent chromatin remodeling complexes (CRCs). Among CRCs, the evolutionarily conserved BAF (BRG1/BRM-associated factor, also known as mammalian SWI/SNF) complexes promote gene expression by increasing access to DNA and thus facilitating transcription, replication, and repair [1]. The BAF complex binds to DNA and histones and effectuates nucleosome displacement to enhance DNA accessibility, thereby initiating the transcription machinery. BAF complexes also act as tumor suppressors as somatic loss-of-function variants in one or more of their components are involved in almost 25% of all cancers [2]. They are organized into three broad subfamilies: canonical BAF (cBAF), polybromo-associated BAF (PBAF), and the GLTSCR1 or GLTSCR1L-containing and BRD9-containing (GBAF) complexes, also known as non-canonical BAF (ncBAF) [2]. The cBAF complex includes the AT-Rich Interaction Domain (ARID) proteins ARID1A and ARID1B, while ARID2 is a component of the PBAF complex [3]. *ARID2* (OMIM*609539), also known as *BAF200*, contains an AT-rich DNA interaction domain consensus located at the N-terminus, followed by RFX, GLN (glutamine-rich region), and two classic C2H2 zinc fingers at the C-terminus, which directly bind to DNA or interact with proteins. *ARID2* is ubiquitously expressed and participates in many biological processes, such as transcriptional regulation, tumorigenesis, development, and differentiation through tissue-specific gene expression and control of cell proliferation [4].

Germline pathogenic variants in genes encoding proteins from the BAF complexes cause a spectra of disorders of which the majority consists of Coffin-Siris (CSS, OMIM#135900) and Nicolaides-Baraitser (NCBRS, OMIM#601358). NCBRS is caused by pathogenic variants in the helicase domain of *SMARCA2* (OMIM*600014) [3]. More recently, de novo *SMARCA2* missense variants clustering outside the helicase domain have been associated with a distinct syndrome with ID and blepharophimosis (OMIM#619293) [5]. In contrast, the phenotypic spectrum of CSS is broader, including developmental delay affecting intellectual and motor functions, hypotonia, growth impairment, coarse features, feeding difficulties in infancy, and hypoplastic to absent fifth distal phalanges of fingernails and toenails [6, 7]. Pathogenic variants in several genes, including *ARID1A* (OMIM*603024), *ARID1B* (OMIM*614556), *SMARCA4* (OMIM*603254), *SMARCB1* (OMIM*601607), *SMARCE1* (OMIM*603111), *SOX11* (OMIM*600898), *DPF2* (OMIM*601671), *SMARCC2* (OMIM*601734), *SOX4* (OMIM*184430), *SMARCD1* (OMIM*60), *BICRA* (OMIM*605690, and *ARID2* (OMIM*609539) are responsible for CSS or CSS-like [8].

In 2015, *ARID2* was first associated to a BAF-related ID syndrome called CSS-6 (OMIM#617808). To date, 26 individuals harboring causative heterozygous *ARID2* loss-of-function variants have been reported [8–18]. The core phenotype described consists of developmental delay, speech disorders, ID, behavior problems, short stature, and various dysmorphic and ectodermal features referring to CSS.

In recent years, high-throughput DNA sequencing has led to the identification of many variants of unknown significance, necessitating further tools for better variant classification. Functional tests can be used to assess the pathogenicity of these variants, including specific methylation signatures called episignature [19]. Because of the interplay between histone positioning, composition, and modification, and DNA methylation at CpG dinucleotides, these biomarkers can sometimes be used to stratify variants in diseases directly or indirectly affecting DNA methylation [20]. ARID2 plays a role in chromatin remodeling. We thus hypothesized that individuals with an ARID2-related disorder (ARID2-RD) may carry a recognizable DNA methylation pattern.

Here, we present evidence for an epigenetic signature associated with ARID2-RD, and we further delineate its phenotypic spectrum, with the report of 27 previously unreported, unrelated individuals harboring *ARID2* loss-of-function variants and a review of 26 previously published individuals.

## Materials and methods

### Individuals

Written informed consent for publication of clinical and genetic data was obtained from all individuals harboring variants of interest in *ARID2* or their legal guardians. Written informed consent for publication of medical photographs was obtained for individuals for whom these are provided. In 22 of 27 individuals, *ARID2* variants were identified using exome or genome sequencing (ES, GS). An intellectual disability gene panel (IDGP) identified one variant. In the remaining 4/27 individuals, *ARID2* deletions were identified using chromosomal microarray analysis (Table S1). The identified *ARID2* variants were deposited in ClinVar (Table S1) [21]. The supplementary data provide details about the methods used for IDGP, ES, GS, and chromosomal microarray analysis.

## Results

### Identification of novel ARID2-RD individuals through genotype-first approach

**Fig. 1:**
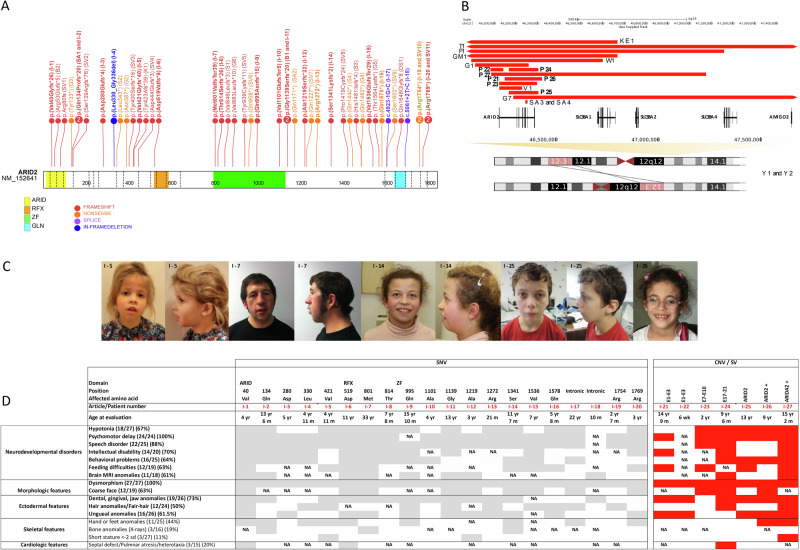
*ARID2* pathogenic variants and associated clinical features. **A** Position of frameshift (in red), nonsense (in orange), splice (in purple), and in-frame deletion (in blue) variants of the cohort and the literature along the *ARID2* sequence. **B** (Micro)deletions identified in the cohort and the literature are represented by the red bars, all affecting *ARID2*. The balanced rearrangement of chromosome 12 in which one of the chromosomal breakpoints was predicted to split *ARID2* is represented in salmon. Genomic positions are according to hg38; ARID2 is on the forward strand. **C** Front and side profile picture of individuals with a PTV or deletion affecting *ARID2*. **D** A table demonstrating the amino acid positions and the clinical findings of *ARID2* variants detected in 27 cohort individuals. When the boxes are colored in gray (for SNV) or red (for CNV/SV), the clinical sign is present; when white, the clinical sign is absent; and when not available, N/A.

Twenty-four affected individuals harboring *ARID2* pathogenic or likely pathogenic variants and interstitial deletions encompassing *ARID2* were recruited through the French network for rare developmental disorders (AnDDI-Rares) and GeneMatcher [22]. Eighteen different single nucleotide variants or indels were identified in the coding sequence of *ARID2* (i.e., 12 frameshift, four nonsense, two splice variants), in addition to six interstitial deletions encompassing *ARID2* (Fig. 1A, B and D). Twenty-one of the twenty-seven (78%) variants were confirmed to have occurred de novo. Inheritance from a healthy parent carrying the pathogenic variant in a mosaic state was detected in two cases (individuals I-21 and I-23), and in I-27 the variant was inherited from the affected mother. In the remaining three cases, one or both parents were unavailable for testing (individuals I-2, I-9, and I-22).

### Pathogenic variants in *ARID2* are associated with a hypermethylated episignature

Next, we sought to investigate whether ARID2-RD individuals with pathogenic variants in *ARID2* are associated with a detectable change in DNA methylation compared to unaffected controls, and if affected individuals showed any stratification correlating with the severity of the disease. The analysis led to the identification of 215 probes (Table S3) whose differentially methylated CpG successfully permitted to separate ARID2 cases (I-2, I-6, I-8, I-10, I-12, I-13, I-14, I-15, I-17, I-18, I-21, I-23, I-25, I-26) from unaffected controls. With unsupervised clustering methods, specifically hierarchical clustering (heatmap) and multidimensional scaling (MDS) methods, we were able to confirm that based on differential methylation from the selected probes, ARID2 cases could be distinguished from controls (Fig. S1). To test the robustness and sensitivity of our ARID2 episignature, we performed 14 rounds of leave-one-out cross-validation using unsupervised hierarchical and MDS clustering methods. All the cases used for testing clustered together with the remaining training cases showing the robustness and sensitivity of the episignature (Fig. S2).

Next, we validated the ARID2 episignature with three additional cases with pathogenic variants in *ARID2* (I-1, I-7, I-11). We performed hierarchical clustering and MDS and confirmed that all the validation cases clustered together with the training cases. With the support vector machine (SVM) classifier model, all the validation samples scored close to one, indicating the presence of the ARID2 episignature (Fig. S3). Using the SVM classifier, we screened all unresolved cases in the EpiSignTM Knowledge Database (EKD), a database including cases and DNA methylation data used by the EpiSignTM classifier. When a clinical case is sent in, and no episignature is found, it gets labeled as unresolved. Every time a new episignature is discovered, we screen all unresolved cases to find matches. With the ARID2 episignature, we found three cases (I-3, I-4, I-24) with an MVP score close to 1, and they all clustered with ARID2 cases in hierarchical clustering and MDS plots. After contacting the clinical centers that submitted the cases, we confirmed that all three individuals carried pathogenic variants in *ARID2* (i.e., one frameshift, one in-frame deletion, and one intragenic deletion encompassing exons 7 through 10).

**Fig. 2:**
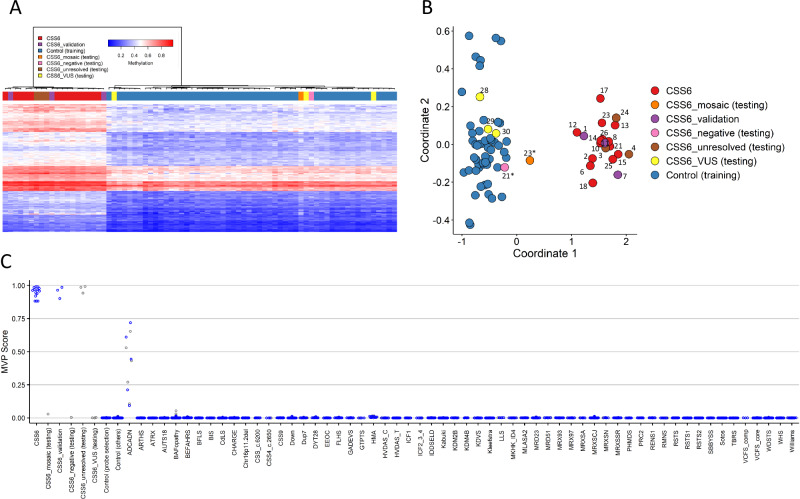
Assessment of the Coffin-Siris syndrome-6 (ARID2) episignature. **A**, **B** Euclidean hierarchical clustering heatmap, each column represents one ARID2 case or control, and each row represents one probe selected for this episignature. This heatmap and multidimensional scaling (MDS) plot shows a clear separation between ARID2 cases (red and purple) used for training and validation from controls (blue). The mosaic case (orange) is shown to map with control cases, one episignature-negative case (pink) is also shown to map with controls, three unresolved cases (brown) are mapping to cases, and four VUS cases (yellow) are mapping with controls. **C** Support Vector Machine (SVM) classifier model. The x-axis represents an episignature on the EpiSign™ classifier, and the y-axis a probability score referred to as a Methylation Variant Pathogenicity score (MVP). This model was trained using the selected ARID2 episignature probes, 75% of controls, and 75% of other neurodevelopmental disorder samples (blue). The remaining 25% of controls and 25% of other disorder samples were used for testing (grey). Plot shows the ARID2 mosaic, negative and VUS cases with a methylation variant pathogenicity (MVP) score close to 0, and the unresolved cases with an MVP score close to 1, similar to the ARID2 training cases, showing the specificity of the classifier and episignature. ADCADN Cerebellar ataxia deafness and narcolepsy syndrome, AUTS18 Susceptibility to autism 18, BEFAHRS Beck-Fahrner syndrome, BFLS Borjeson–Forssman– Lehmann syndrome, BISS Blepharophimosis intellectual disability SMARCA2 syndrome, CdLS Cornelia de Lange syndrome, CHARGE CHARGE syndrome, Chr16p11.2del Chromosome 16p11.2 deletion syndrome, CSS Coffin–Siris syndrome, CSS4 Coffin-Siris syndrome 4, CSS9 Coffin–Siris syndrome 9, Down Down syndrome, Dup7 7q11.23 duplication syndrome, DYT28 Dystonia 28, EEOC Epileptic encephalopathy-childhood onset, FLHS Floating Harbour syndrome, GTPTS Genitopatellar syndrome, HMA Hunter McAlpine craniosynostosis syndrome, HVDAS Helsmoortel–van der Aa syndrome, ICF Immunodeficiency-centromeric instability-facial anomalies syndrome, IDDSELD Intellectual developmental disorder with seizures and language delay, Kabuki Kabuki syndrome, KDVS Koolen-De Vries syndrome, Kleefstra Kleefstra syndrome, LLS Luscan-Lumish syndrome, MKHK Menke Hennekam syndrome, MLASA2 Myopathy lactic acidosis and sideroblastic anemia 2, MRD23 Intellectual developmental disorder 23, MRD51 Intellectual developmental disorder 51, MRX93 Intellectual developmental disorder X-linked 93, MRX97 Intellectual developmental disorder X-linked 97, MRXSA Intellectual developmental disorder X-linked syndromic Armfield type, MRXSCH Intellectual developmental disorder X-linked syndromic Christianson type, MRXSCJ Intellectual developmental disorder X-linked syndromic Claes-Jensen type, MRXSN Intellectual developmental disorder X-linked syndromic Nascimento type, MRXSSR Intellectual developmental disorder X-linked syndromic Snyder–Robinson type, PHMDS Phelan–McDermid syndrome, PRC2 PRC2 complex (Weaver and Cohen-Gibson) syndrome, RENS1 Renpenning syndrome, RMNS Rahman syndrome, RSTS Rubinstein–Taybi syndrome, SBBYSS Ohdo syndrome, Sotos Sotos syndrome, TBRS Tatton–Brown– Rahman syndrome, WDSTS Wiedemann–Steiner syndrome, WHS Wolf-Hirschhorn syndrome, Williams Williams syndrome.

One of the most interesting utilities of episignatures is that they assist in reclassifying variants of uncertain significance (VUS). Therefore, we evaluated the episignature associated with two phenotypically unaffected individuals bearing mosaic variants (I-21* and I-23*) which are parents of two affected cases included in our cohort, three VUS cases (I-28, I-29, I-30) identified through GeneMatcher and the AnDDI-Rares network, and three unresolved cases (I-3, I-4, I-24), and compared them to the training cohort (discovery + validation cases) for assessment. The heatmap shows a clear separation between the ARID2 discovery and validation cases used for training and the controls (Fig. 2A). The two healthy mosaic individuals and the three VUS cases clustered with controls. The three unresolved affected individuals clustered with the ARID2 cases. The MDS plot shows the same results as the heatmap (Fig. 2B). The mosaic case (I-23*) is shown to map between cases and controls in MDS, although closer to controls. The SVM classifier model was trained using the selected ARID2 episignature probes, 75% of controls, and 75% of other neurodevelopmental disorder samples. The remaining 25% of controls and 25% of other disorder samples were used for testing. The plot shows the ARID2 mosaic negative and VUS cases with an MVP score close to 0 and the unresolved cases with MVP scores close to 1, similar to the ARID2 training cases, showing the specificity of the classifier and episignature (Fig. 2C).

However, the MVP classifier is also showing that cases with an autosomal dominant cerebellar ataxia, deafness, and narcolepsy (ADCADN) (OMIM #604121) episignature are responding on the ARID2 episignature based on the MVP score being higher than 0, some as high as 0.75. This could be explained by the effect size on DNA methylation across a significant proportion of the CpG probes due to DNMT1 involvement in the ADCADN disorder. Therefore, this episignature showed the largest changes in methylation between cases and controls and overlapped with several other cohorts [23]. However, this does not affect our ARID2 episignature as there is still a separation between controls, ARID2 cases, and ADCADN cases when the cases are plotted together using unsupervised clustering methods (Figure S4).

### Differentially methylated regions (DMRs)

We found 12 DMRs (Table S3) that were all hypermethylated, which is in line with the discovered episignature. Two of the DMRs were located on each of chromosomes 22, 17, 10, 7, and 3, and one DMR each on chromosomes 6 and 1. One of the DMRs overlapped *MC4R* (OMIM *155541), which is associated with autosomal dominant and recessive obesity. Another DMR overlapped *TRAK1* (OMIM *608112), which is associated with autosomal recessive developmental and epileptic encephalopathy.

### Overlap of CSS-6 Genome-Wide DNA Methylation Profile with Other Neurodevelopmental Disorders on EpiSign™

**Fig. 3:**
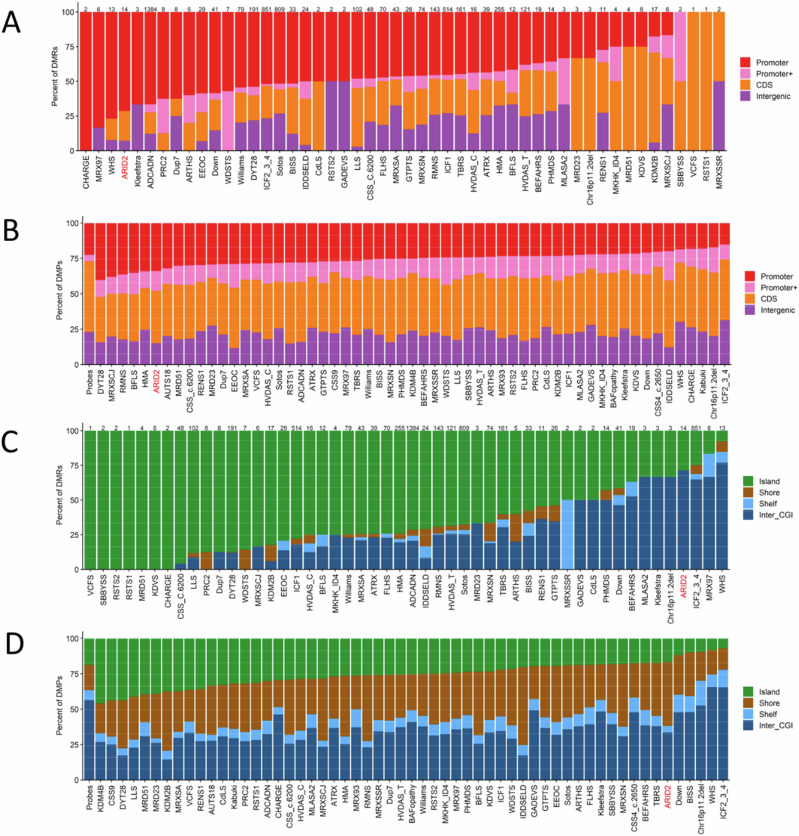
Differentially methylated probes (DMPs) annotated in the context of CpG islands and genes. **A** DMRs in relation to genes. **B** DMPs in relation to genes. **C** DMRs in CpG islands and (**D**) DMPs in CpG island. Promoter, 0–1 kb upstream of the transcription start site (TSS); Promoter + , 1–5 kb upstream of the TSS; CDS, coding sequence; Intergenic, all other regions of the genome. Island, CpG islands; Shore, within 0–2 kb of a CpG island boundary; shelf, within 2-4 kb of a CpG island boundary; Inter_CGI, all other regions in the genome. The Probes column in both (b,d) represents the background distribution determined in the Levy et al. [9] study of all array probes after initial filtering and used as input for DMP analysis.

Next, we annotated the genomic location of the DMPs (differentially methylated probes) and DMRs (differentially methylated regions) in relation to the CpG islands and genes. We found that the 12 DMRs were mostly found in the promotor regions in relation to genes of which 71% in the inter_CGI sites, and 29% in the islands in relation to CpG islands (Figs. 3A and 2C). DMPs are mostly found in the CpG island shores (45%), inter_CGI (33%), islands (17%), and shelfs (5%), and in relation to genes they are mostly found in the promotor regions (Fig. 3B, D).

**Fig. 4:**
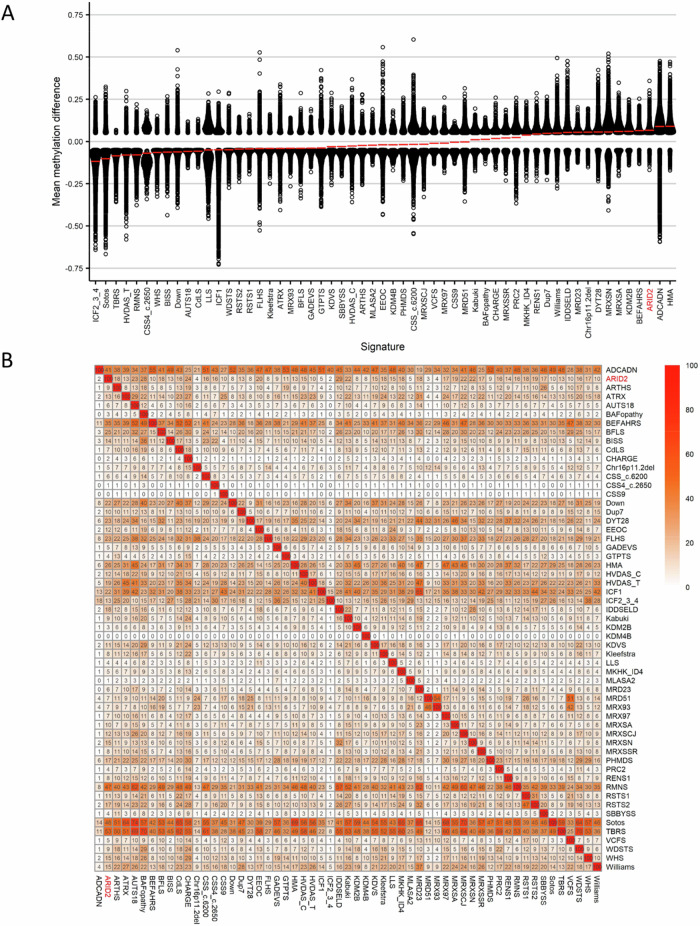
Relationships between the ARID2 cohort and 56 other EpiSign™ disorders. **A** Global methylation profiles of all differentially methylated probes (DMPs, FDR < 0.05) for each cohort, sorted by mean methylation. Each circle represents one probe, and red lines show the mean methylation. **B** Heatmap showing the percentage of probes shared between each paired cohort. Colors indicate the percentage of the y-axis cohort’s probes that are also found in the x-axis cohort’s probes.

Finally, we investigated the overlap of the *ARID2* genome-wide DNA methylation changes in cases with pathogenic *ARID2* variants and the other 56 episignatures described by Levy et al. [24]. Clustering analyses were performed using the top 500 DMPs for each cohort, for cohorts with less than 500 DMPs, the total number of DMPs was used to assess the similarity in genome-wide methylation profiles. We detected a predominant hypermethylation profile (Fig. 4A). Levy et al. [24] showed that 66% (*n* = 37) of the episignatures showed hypomethylation and 34% (*n* = 19) hypermethylation in the other 56 EpiSignTM disorders. *ARID2* had the highest percent of DMPs overlapping with Intellectual developmental disorder with seizures and language delay (IDDSELD) (29%, *SETD1B*), BAFopathy (28%, including *ARID1A*, *ARID1B*, *SMARCB1*, *SMARCA2*, *SMARCA4*), CHARGE (24%, *CHD7*), Intellectual developmental disorder with autism and macrocephaly (IDDAM) (23%, *CHD8*), Kabuki syndrome (22%, *KMT2D*), Intellectual developmental disorder, X-linked 93 (*MRX93*) (22%, *BRWD3*) and Intellectual developmental disorder, X-linked 97 (*MRX97*) (22%, *ZNF711*) (Figs. 4B and  S5).

**Fig. 5:**
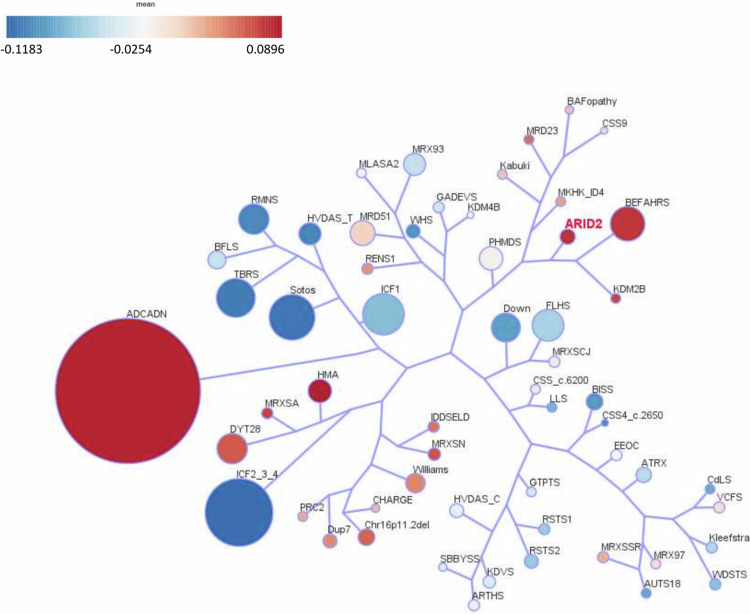
Tree and leaf visualization of Euclidean clustering of all 57 cohorts using the top n DMPs for each group, where *n* = min (# of DMPs, 500). Cohort samples were aggregated using the median value of each probe within a group. A leaf node represents a cohort, with node sizes illustrating relative scales of the number of selected DMPs for the corresponding cohort, and node colors are indicative of the global mean methylation difference.

To investigate the relation between the 57 cohorts, we showed the DMP overlap and the hypomethylation or hypermethylation levels with a binary tree where each node corresponds to a cohort. Here, we observed that ARID2 clustered in a hypermethylation branch close to Beck-Fahrner syndrome (BEFAHRS; *TET3*) and Kabuki syndrome (*KMT2D*) (Fig. 5). The DMPs that are shared between those cohorts give us an indication that there might be common underlying biological processes that can impact each disorder due to the overlap in functional consequences to the epigenome.

### Clinical results

Our clinical cohort comprised 27 individuals harboring pathogenic variants in *ARID2* (i.e., 24 individuals identified through AnDDI-Rares and GeneMatcher, and three individuals from the EKD). All individuals in this series presented with psychomotor delay, and 67% had hypotonia. Most had speech disorders (88%), behavioral abnormalities (64%), ID (70% in total, of which 79% mild and 21% moderate), and ectodermal features with dental, hair, and nail anomalies (73%, 50%, and 61.5%). Few have short stature (15%). The clinical characteristics of this series are summarized in Table 1, Fig. 1C, and given in detail in Table S1. Shared facial features included a coarse face, wide mouth, down-slanted palpebral fissures, and low-set, posteriorly rotated ears (Fig. 1D). Detailed reports, including variant classification scores, are available in Table S1. We did not identify a clear correlation between symptom severity and variant location (Fig. 1A; D, Table S4).

**Table 1:** Clinical findings and their distribution for 49 new and already published individuals with ARID2-related disorder.

Clinical Finding ^a^	This Series	Literature	Total
Sex (males/females, *n* [%])(*N* = 27 + 26)	13 (*48*)/14 (*52*)	17 (*65*)/9 (*35*)	30 (*57*)/23 (*43*)
**Neurodevelopmental disorders**			
Hypotonia *n*(%) (*N* = 27 + 20)	18 (*67*)	14 (*70*)	32 (*68*)
Psychomotor delay *n*(%) (*N* = 24 + 25)	24 (*100*)	24 (*96*)	48 (*98*)
Speech disorder *n*(%) (*N* = 25 + 15)	22 (*88*)	13 (*87*)	35 (*87.5*)
Intellectual disability *n*(%) (*N* = 20 + 23)	14 (*70*)	22 (*96*)	36 (83)
Epilepsy *n*(%) (*N* = 26 + 24)	2 (*8*)	2 (*8*)	4 (*8*)
Brain MRI anomalies^b^ *n*(%) (*N* = 18 + 13)	11 (*61*)	8 (*62*)	19 (*61*)
**Behavioral problems** *n*(%) (*N* = 25 + 23)	16 (*64*)	15 (*65*)	31 (*64.5*)
Autism spectrum disorder *n*(%) (*N* = 25 + 23)	0 (*0*)	2 (*9*)	2 (*4*)
Attention deficit/hyperactivity disorder(%) (*N* = 25 + 23)	10 (40)	7 (*30*)	17 (*35*)
Low frustration tolerance *n*(%) (*N* = 25 + 23)	5 (20)	3 (*13*)	8 (*17*)
Aggressive behavior *n*(%) (*N* = 25 + 23)	3 (12)	3 (*13*)	6 (*12.5*)
Anxiety disorder *n*(%) (*N* = 25 + 23)	3 (12)	4 (*17*)	7 (*15*)
**Feeding difficulties** *n*(%) (*N* = 19 + 26)	12 (*63*)	8 (*31*)	20 (*44*)
**Morphologic features**			
Facial features*n*(%) (*N* = 27 + 26)	27 (*100*)	25 (*96*)	52 (*98*)
Coarse face *n*(%) (*N* = 19 + 21)	12 (*63*)	15 (*71*)	27 (*67.5*)
**Ophtalmologic Features**			
Ptosis *n*(%) (*N* = 23 + 23)	2 (*9*)	3 (*13*)	5 (*11*)
Myopia *n*(%) (*N* = 23 + 18)	4 (*17*)	6 (*33*)	10 (*24*)
Hyperopia *n*(%) (*N* = 23 + 17)	6 (*26*)	3 (*18*)	9 (*22.5*)
Strabismus *n*(%) (*N* = 23 + 18)	5 (*22*)	7 (*39*)	12 (*29*)
Astigmatism *n*(%) (*N* = 23 + 17)	2 (*9*)	1 (*6*)	3 (*7.5*)
**Deafness** *n*(%) (*N* = 20 + 12)	2 (*10*)	3 (*25*)	5 (*16*)
**Ectodermal features**			
Dental, gingival, jaw anomalies *n*(%) (*N* = 26 + 25)	19 (*73*)	6 (*24*)	25 (*49*)
Hair anomalies/Fair-hair *n*(%) (*N* = 24 + 20)	12 (50)	10 (*50*)	22 (*50*)
Nail anomalies *n*(%) (*N* = 26 + 21)	16 (*61.5*)	8 (*38*)	24 (*51*)
**Skeletal features**			
Bone anomalies (X-rays) *n*(%) (*N* = 16 + 19)	3 (*19*)	6 (*32*)	9 (*26*)
Hand or feet anomalies *n*(%) (*N* = 25 + 26)	11 (*44*)	15 (*58*)	26 (*51*)
Short stature <-2 sd *n*(%) (*N* = 27 + 25)	4 (*15*)	15 (*60*)	19 (*36.5*)
**Cardiologic features** *n*(%) (*N* = 15 + 15)	3 (*20*)	4 (*27*)	7 (*23*)

^a^The number of responders is detailed for every feature, and their frequencies/distributions were calculated according to that number, e.g., for hypotonia, (*N* = 27 + 20) means data were available for 27 individuals from our series and 20 individuals from the literature and 18(67) means 18 individuals in our series presented with hypotonia, which represents 67% of individuals in our series.

^b^MRI brain findings include cerebral atrophy, ventriculomegaly, or white matter hyperintensities.

In the literature [8–13, 15–18] (Table 1, Table S4), the *ARID2* associated phenotype mainly consists of psychomotor delay (96%), hypotonia (70%), speech disorders (87%), behavior problems (65%), variable range of ID from mild to severe (96%), ectodermal features (hair 50%, and nail anomalies 38%), and few dental anomalies (24%). Short stature is frequent (60%). facial features (96%) and coarse face (71%) are also often reported.

Overall, the clinical assessment of the newly identified individuals allowed us to define the phenotypic spectrum of ARID2-RD, suggesting that ID is often mild, and when absent, individuals without an ID seem to have learning disabilities or a heterogenous cognitive profile. Short stature is less frequently observed than previously reported (range −2.9 to 2.38 SD). Also, we did not note any apparent correlation between the severity of the disease and the type/localization of the variants (Fig. 1A; D).

## Discussion

ARID2-RD is a rare monogenic disorder. Our study increases the total number of individuals reported with *ARID2* pathogenic variants from 26 to 53 and further delineates the phenotypic spectrum of the *ARID2* associated CSS 6 to be rather mild in many cases. There was no apparent genotype-phenotype correlation deducible despite the larger patient number. Consistent with previous reports, we observed loss-of-function as pathomechanism and we provide evidence of a unique DNA methylation episignature for ARID2-RD.

### Molecular description

All variants reported here and in the literature (i.e., 30 frameshifts, 13 nonsense, one in-frame deletion, and two splice variants) are distributed throughout the gene. The majority of the variants result in a premature termination codon (PTC). They are predicted to result in nonsense-mediated decay and hence loss-of-function, as is the assumed pathomechanism for all *ARID2* pathogenic variants reported. This is consistent with the “probability of being loss‐of‐function intolerant” (pLI) score of 1 [25] and the loss-of-function observed/expected upper bound fraction (LOEUF) score of 0.225, reflecting the high intolerance of this gene to loss-of-function variants. The lack of a clear genotype-phenotype correlation observed in our series and the literature (Fig. 1D, Table S1, Table S4) is an additional indirect argument indicating that the deleteriousness of *ARID2* variants results from haploinsufficiency after nonsense-mediated mRNA decay. Likewise*, ARID2* haploinsufficiency is the consequence of 17 chromosomal rearrangements encompassing *ARID2* reported so far, including the seven *ARID2* deletions in our study (Fig. 1B–D) [9–11, 15, 17, 18, 26].

Similarly, we do not observe a contiguous gene deletion phenotype but a ARID2 haploinsufficiency phenotype. Genetic background may partially account for phenotypic variability, including ID differences, where polygenic contributions can impact global IQ scores [27]. Moreover, multiple genetic diseases could also contribute to phenotypic variability, as exemplified by the genetic findings in individual 11 of this study and individual 1 published by Bramswig et al. (listed as individual B1 in Fig. 1A and Table S4) [28]. They harbored the same pathogenic variant (Fig. 1B, Table S1); however, individual B1 had also a de novo nonsense variant in the *TRIM8* gene explaining a more severe ID. On average, multiple diseases are expected in 4.3% of diagnosed patients with already identified genetic disorders [29, 30].

### Clinical description

The phenotype of ARID2-related disorders primarily involves neurodevelopmental issues that have been consistently observed in affected individuals (Table S1) [8–12, 14–18, 26, 28]. However, individuals in our group had less severe intellectual disability (ID) compared to previously reported cases. Importantly, three individuals in our group (I-5, I-17, I-25) did not exhibit ID, but they did show signs of developmental delay, learning disability, a heterogenous cognitive profile, or speech disorder. This emphasizes the significance of early intervention. This variability did not seem related to the variant’s position in the gene. Neither our cohort nor the literature reported any specific MRI abnormalities. Cerebral atrophy, ventriculomegaly, or white matter hyperintensities are described.

It is important to pay special attention to behavioral problems as they are common in this disorder, with 64.5% of cases suffering from them according to Gazdagh et al. [10]. In our study, and generally, the most common disorder was ADHD (40% and 35%, respectively). However, low frustration tolerance (20% and 17%) and anxiety disorders (12% and 15%) were also reported.

Individuals with *ARID2* loss-of-function variants share dysmorphic facial features, including coarse facies (67.5%), down-slanting palpebral fissures, low-set, and posteriorly rotated ears, as well as relatively short nose and long philtrum [28]. (Fig. 1C, Table S1). Several articles mentioned a resemblance between the dysmorphic facial features linked to *ARID2* and those in RASopathies [11, 15], such as Noonan syndrome, as in I-7 of this series (Fig. 1C).

Ectodermal abnormalities in CSS, such as specific nail abnormalities (i.e., small nails on the 5th finger or toe), are found in half of individuals with *ARID2* variants. Note also that 49% of all reported individuals have dental, gingival, and jaw abnormalities such as gingival overgrowth, persistence of primary teeth, tooth malposition, macrodontia, and retrognathia (Table 1, Fig. 1C, D).

Feeding problems, which affect 63% of our cohort and 44% of individuals, are also a common feature of this disorder. On the other hand, short stature is less frequent in our cohort (11%) than reported in the literature (60%) [8–12, 14–18] (Table 1, Table S1). These relatively low percentages indicate that growth abnormalities are not consistent in ARID2-related disorders.

### Epigenetic signature

We identified a robust and sensitive episignature for ARID2-RD, consisting of the methylation profile in 215 differentially methylated CpG probes, which allows to discriminate individuals with ARID2-RD vs. unaffected controls. The ADCADN episignature that already overlaps with several other cohorts [23] is also responding on the ARID2 episignature, but does not affect the specificity of our ARID2 episignature. An episignature is helpful to 1) reclassify VUS, which are increasing in number due to high throughput sequencing techniques [31]. ; 2) screen unresolved cases to find matches as demonstrated by the cases of I-3 (c.836_839dup, p.Asp280Glufs*4), I-4 (c.988_1008del, p.(Leu330_Gly336del)), and I-24 (g.(?_45891780)_(45905075_?)del). 3) Detect variant in a mosaic state as shown in I-23* (12 :(46123620-46298861)x1).

The gnomAD constraint score for missense variants is rather high (Z = 4.49) with three regions particularly depleted of predicted deleterious missense variants at the N-terminal (amino acid 1–455, regional missense constraint (RMC) = 0.46, *p* value = 2.57 ×10–17) and the C-terminal (aa 1634–1710, RMC = 0.29, *p*-Value = 1.32 ×10−5; and aa 1758–1835, RMC = 0.21, *p*-Value = 1.03 ×10−6) part of the protein, according to Decipher. These metrics suggest that deleterious missense variants with loss-of-function effect may also be causative for ARID2-RD. However, no such variants have been identified to date. In our cohort four missense VUS in I-28 (c.1150 G > A; p.(Ala384Thr) and c.4585 G > A; p.(Gly1529Arg)), I-29 (c.484 G > T; p.(Val162Leu)), and I-30 (c.335 A > C; p.(Glu112Ala)) were negative for being associated with a loss-of-function effect. Indeed, a negative episignature result does not rule out the implication variants in a different (e.g. gain-of-function) ARID2-related disease. However, in our study other criteria contributed to the reclassification of the VUS in I-28 (c.1150 G > A; p.(Ala384Thr) and c.4585 G > A; p.(Gly1529Arg)), I-29 (c.484 G > T; p.(Val162Leu)), and I-30 (c.335 A > C; p.(Glu112Ala)) variants as being nonpathogenic such as the inheritance from an unaffected parent (c.1150 G > A and c.4585 G > A identified in individual I-28 were inherited from the healthy father and healthy mother respectively, c.484 G > T identified in individual I-29 was inherited from the healthy father; c.335 A > C identified in individual I-30 was inherited from the healthy father), or the predicted impact on the protein was likely_benign and ambiguous for I-28, likely_pathogenic for I-29 and ambiguous for I-30 according to Alphamissense score [32].

We found 12 DMRs that were hypermethylated. One of the DMRs overlapped with *MC4R* associated with autosomal dominant and recessive obesity, a phenotype not described in young individual with ARID2-RD. However, it may be a phenotype that only develops with time, and it would be interesting to monitor weight trends in individuals with ARID2-RD. Another DMR overlapped *TRAK1* associated with autosomal recessive developmental and epileptic encephalopathy. Further investigation is needed to study the possible involvement of these regions and genes in the pathological mechanisms of ARID2-RD.

Finally, when we investigated the overlap of the ARID2 genome-wide DNA methylation changes in individuals with pathogenic *ARID2* variants and other 56 established episignatures, we found an overlap with Intellectual developmental disorder, seizures, and language delay (IDDSELD), BAFopathy, CHARGE, Intellectual developmental disorder with autism and macrocephaly (AUTS18), Kabuki syndrome, Intellectual developmental disorder, X-linked 93 (MRX93) and Intellectual developmental disorder, X-linked 97 (MRX97). These neurodevelopmental syndromes share features with ARID2-RD, such as hypotonia, psychomotor delay, speech disorders, ID, and behavioral disorders. And some of them share morphologic features such as BAFopathy. These findings indicate possible underlying pathophysiological processes associated with ARID2-RD, such as post-translational binding of a ubiquitin-like protein shared by some of the proteins (*ARID2, CHD8, KMT2D, TET3*, and *ZNF711*) carrying a specific motif. These results warrant further investigations.

In conclusion, our study reviewed features of 53 individuals with ARID2-RD, including 27 unreported cases. The pathomechanism is *ARID2* haploinsufficiency and there is no clear relationship between genotype and phenotype. We have also identified a unique DNA methylation pattern, which may aid in the diagnosis of ARID2-RD in the future. Additionally, our findings broaden the clinical spectrum of this disorder by describing milder phenotypes than previously reported, specifically regarding cognitive abilities and growth.

## Supplementary information

Supplemental data

Supplemental tables S1-S4

## Data Availability

The raw DNA methylation data for the samples are unavailable due to institutional and ethical restrictions. References sites web: http://anddi-rares.org/https://genematcher.org/https://pecan.stjude.cloud/variants/proteinpaint.

## References

[1] Tang L, Nogales E, Ciferri C. Structure and function of SWI/SNF chromatin remodeling complexes and mechanistic implications for transcription. Prog Biophys Mol Biol. 2010;102:122–8. 20493208 10.1016/j.pbiomolbio.2010.05.001 PMC2924208

[2] Mittal P, Roberts CWM. The SWI/SNF complex in cancer — biology, biomarkers and therapy. Nat Rev Clin Oncol. 2020;17:435–48. 32303701 10.1038/s41571-020-0357-3 PMC8723792

[3] Raab JR, Resnick S, Magnuson T. Genome-Wide Transcriptional Regulation Mediated by Biochemically Distinct SWI/SNF Complexes. PLoS Genet. 2015;11:e1005748. 10.1371/journal.pgen.1005748 PMC4699898 26716708

[4] St. Pierre R, Kadoch C. Mammalian SWI/SNF complexes in cancer: emerging therapeutic opportunities. Curr Opin Genet Dev. 2017;42:56–67. 28391084 10.1016/j.gde.2017.02.004 PMC5777332

[5] Cappuccio G, Sayou C, Tanno PL, Tisserant E, Bruel A-L, Kennani SE, et al. De novo SMARCA2 variants clustered outside the helicase domain cause a new recognizable syndrome with intellectual disability and blepharophimosis distinct from Nicolaides-Baraitser syndrome. Genet Med J Am Coll Med Genet. 2020;22:1838–50. 10.1038/s41436-020-0898-y 32694869

[6] Coffin GS. Mental retardation with absent fifth fingernail and terminal phalanx. Arch Pediatr Adolesc Med. 1970;119:433. 10.1001/archpedi.1970.02100050435009 5442442

[7] Tsurusaki Y, Okamoto N, Ohashi H, Mizuno S, Matsumoto N, Makita Y, et al. Coffin–Siris syndrome is a SWI/SNF complex disorder. Clin Genet. 2014;85:548–54. 23815551 10.1111/cge.12225

[8] Schmetz A, Lüdecke H-J, Surowy H, Sivalingam S, Bruel A-L, Caumes R, et al. Delineation of the adult phenotype of Coffin-Siris syndrome in 35 individuals. Hum Genet. 2024;143:71–84. 38117302 10.1007/s00439-023-02622-5

[9] Gallego M, Barreiro C, Pérez, M, Arroyo H, Menehem J. A New Case of Interstitial 12q Deletion. Int Pediatr. 2000;15:37–40.

[10] Gazdagh G, Blyth M, Scurr I, Turnpenny PD, Mehta SG, Armstrong R, et al. Extending the clinical and genetic spectrum of ARID2 related intellectual disability. A case series of 7 patients. Eur J Med Genet. 2019;62:27–34. 29698805 10.1016/j.ejmg.2018.04.014

[11] Kang E, Kang M, Ju Y, Lee SJ, Lee YS, Woo DC, et al. Association between ARID2 and RAS-MAPK pathway in intellectual disability and short stature. J Med Genet. 2021;58:767–77. 10.1136/jmedgenet-2020-107111 33051312

[12] Khazanchi R, Ronspies CA, Smith SC, Starr LJ. Patient with anomalous skin pigmentation expands the phenotype of ARID2 loss-of-function disorder, a SWI/SNF-related intellectual disability. Am J Med Genet A. 2019;179:808–12. 30838730 10.1002/ajmg.a.61075

[13] Schrier Vergano SA. ARID2, a milder cause of Coffin-Siris Syndrome? Broadening the phenotype with 17 additional individuals. Am J Med Genet A. 2024;194:e63540. 10.1002/ajmg.a.63540 38243407

[14] Shang L, Cho MT, Retterer K, Folk L, Humberson J, Rohena L, et al. Mutations in ARID2 are associated with intellectual disabilities. Neurogenetics. 2015;16:307–14. 26238514 10.1007/s10048-015-0454-0

[15] Tonoki H, Saitoh S, Kobayashi K. Patient with del(12)(q12q13.12) manifesting abnormalities compatible with Noonan syndrome. Am J Med Genet. 1998;75:416–8. 9482650 10.1002/(sici)1096-8628(19980203)75:4<416::aid-ajmg13>3.0.co;2-r

[16] Van Paemel R, De Bruyne P, van der Straaten S, D’hondt M, Fränkel U, Dheedene A, et al. Confirmation of an ARID2 defect in SWI/SNF-related intellectual disability. Am J Med Genet A. 2017;173:3104–8. 28884947 10.1002/ajmg.a.38407

[17] Weng Y, Luo X, Hou L. Deletion at 12q12 increases the risk of developmental delay and intellectual disability. Ann Hum Genet. 2018;82:482–7. 30155906 10.1111/ahg.12279 PMC6220791

[18] Yatsenko SA, del Valle Torrado M, Fernandes PH, Wiszniewska J, Gallego M, Herrera J, et al. Molecular characterization of a balanced rearrangement of chromosome 12 in two siblings with Noonan syndrome. Am J Med Genet A. 2009;149A:2723–30. 19938085 10.1002/ajmg.a.33112

[19] Aref-Eshghi E, Bend EG, Colaiacovo S, Caudle M, Chakrabarti R, Napier M, et al. Diagnostic utility of genome-wide DNA methylation testing in genetically unsolved individuals with suspected hereditary conditions. Am J Hum Genet. 2019;104:685–700. 30929737 10.1016/j.ajhg.2019.03.008 PMC6451739

[20] Kerkhof J, Rastin C, Levy MA, Relator R, McConkey H, Demain L, et al. Diagnostic utility and reporting recommendations for clinical DNA methylation episignature testing in genetically undiagnosed rare diseases. Genet Med Off J Am Coll Med Genet. 2024;26:101075. 10.1016/j.gim.2024.101075 38251460

[21] Landrum MJ, Lee JM, Benson M, Brown GR, Chao C, Chitipiralla S, et al. ClinVar: improving access to variant interpretations and supporting evidence. Nucleic Acids Res. 2018;46:D1062–7. 29165669 10.1093/nar/gkx1153 PMC5753237

[22] Sobreira N, Schiettecatte F, Valle D, Hamosh A. GeneMatcher: a matching tool for connecting investigators with an interest in the same gene. Hum Mutat. 2015;36:928–30. 26220891 10.1002/humu.22844 PMC4833888

[23] Aref-Eshghi E, Kerkhof J, Pedro VP, Groupe DIF, Barat-Houari M, Ruiz-Pallares N, et al. Evaluation of DNA methylation episignatures for diagnosis and phenotype correlations in 42 mendelian neurodevelopmental disorders. Am J Hum Genet. 2020;106:356–70. 32109418 10.1016/j.ajhg.2020.01.019 PMC7058829

[24] Levy MA, McConkey H, Kerkhof J, Barat-Houari M, Bargiacchi S, Biamino E, et al. Novel diagnostic DNA methylation episignatures expand and refine the epigenetic landscapes of Mendelian disorders. HGG Adv. 2022;3:100075. 35047860 10.1016/j.xhgg.2021.100075 PMC8756545

[25] Lek M, Karczewski KJ, Minikel EV, Samocha KE, Banks E, Fennell T, et al. Analysis of protein-coding genetic variation in 60,706 humans. Nature. 2016;536:285–91. 27535533 10.1038/nature19057 PMC5018207

[26] Failla P, Romano C, Reitano S, Benedetto DD, Grillo L, Fichera M, et al. 12q12 deletion: A new patient contributing to genotype–phenotype correlation. Am J Med Genet A. 2008;146A:1354–7. 18412123 10.1002/ajmg.a.32280

[27] Kurki MI, Saarentaus E, Pietiläinen O, Gormley P, Lal D, Kerminen S, et al. Contribution of rare and common variants to intellectual disability in a sub-isolate of Northern Finland. Nat Commun. 2019;10:410. 30679432 10.1038/s41467-018-08262-y PMC6345990

[28] Bramswig NC, Caluseriu O, Lüdecke H-J, Bolduc FV, Noel NCL, Wieland T, et al. Heterozygosity for ARID2 loss-of-function mutations in individuals with a Coffin–Siris syndrome-like phenotype. Hum Genet. 2017;136:297–305. 28124119 10.1007/s00439-017-1757-z

[29] Posey JE, Harel T, Liu P, Rosenfeld JA, James RA, Coban Akdemir ZH, et al. Resolution of disease phenotypes resulting from multilocus genomic variation. N. Engl J Med. 2017;376:21–31. 27959697 10.1056/NEJMoa1516767 PMC5335876

[30] Balci TB, Hartley T, Xi Y, Dyment DA, Beaulieu CL, Bernier FP, et al. Debunking Occam’s razor: Diagnosing multiple genetic diseases in families by whole-exome sequencing. Clin Genet. 2017;92:281–9. 28170084 10.1111/cge.12987

[31] Sadikovic B, Levy MA, Kerkhof J, Aref-Eshghi E, Schenkel L, Stuart A, et al. Clinical epigenomics: genome-wide DNA methylation analysis for the diagnosis of Mendelian disorders. Genet Med J Am Coll Med Genet. 2021;23:1065–74. 10.1038/s41436-020-01096-4 PMC8187150 33547396

[32] Cheng J, Novati G, Pan J, Bycroft C, Žemgulytė A, Applebaum T, et al. Accurate proteome-wide missense variant effect prediction with AlphaMissense. Science. 2023;381:eadg7492. 37733863 10.1126/science.adg7492

