# Supplementary material for: ARID2-related disorder: further delineation of the clinical phenotype of 27 novel individuals and description of an epigenetic signature
Source: Eur J Hum Genet. 2025 Mar 5;33(11):1422–31. doi: 10.1038/s41431-025-01798-w (PMC12583565; doi:10.1038/s41431-025-01798-w)
Supplement: Supplementary file 1 — Supplemental data [file 41431_2025_1798_MOESM1_ESM.docx]

**Supplementary Data**

**ARID2-related disorder: Further delineation of the clinical phenotype of 27 novel individuals and description of an epigenetic signature**

Clara Houdayer ^1,2#^, Kathleen Rooney ^3,4#^, Liselot van der Laan ^5#^, Céline Bris ^1,2^, Mariëlle Alders ^5^, Angela Bahr ^6^, Giulia Barcia ^7^, Clarisse Battault ^1,2^, Anais Begemann ^6^, Dominique Bonneau ^1,2^, Antoine Bonnevalle ^8^, Aicha Boughalem ^9^, Alice Bourges ^1,2^, Marie Bournez ^10,11^, Ange-Line Bruel ^10,11^, Daniela Buhas ^12,13^, Floriane Carallis ^14^, Benjamin Cogné ^15,16^, Valérie Cormier-Daire ^7^, Julian Delanne ^10,11^, Tanguy Demaret ^17^, Anne-Sophie Denommé-Pichon ^10,11^, Julie Desir ^17^, Christèle Dubourg ^18^, Mélanie Fradin ^18^, David Geneviève ^19,20^, Himanshu Goel ^21^, Alice Goldenberg ^8^, Karen W Gripp ^22^, Agnès Guichet ^1,2^, Anne Guimier ^7^, Adeline Jacquinet ^23^, Boris Keren ^24^, Louis Legoff ^1^, Michael A. Levy ^3^, Haley McConkey ^3^, Bryce A. Mendelsohn ^25^, Cyril Mignot ^26^, Vincent Milon ^1^, Mathilde Nizon ^15,16^, Beatrice Oneda ^6^, Laurent Pasquier ^18^, Olivier Patat ^27^, Christophe Philippe ^10,11^, Vincent Procaccio ^1,2^, Rebecca Procopio ^22^, Clément Prouteau ^1^, Thomas Rambaud ^14^, Anita Rauch ^6^, Raissa Relator ^3^, Sophie Rondeau ^7^, Gijs W.E. Santen ^27^, Jennifer Schleit ^28^, Arthur Sorlin ^10,11^, Katharina Steindl ^6^, Matt Tedder ^29^, Marine Tessarech ^1,2^, Frédéric Tran Mau-Them ^10,11^, Detlef Trost ^9^, Pleuntje J. Van der Sluijs ^28^, Marie Vincent ^15,16^, Sandra Whalen ^24^, Christel Thauvin-Robinet ^10,11^, Bertrand Isidor ^15,16^, Bekim Sadikovic ^3,4#^, Antonio Vitobello ^10,11#^, Estelle Colin ^1,2#^

# Contributed equally

^1^ Service de Génétique Médicale, CHU d’Angers, Angers, France

^2^ Univ Angers, [CHU Angers], INSERM, CNRS, MITOVASC, SFR ICAT, F-49000 Angers, France

^3^ Verspeeten Clinical Genome Centre, London Health Science Centre, London, ON, Canada

^4^ Department of Pathology and Laboratory Medicine, Western University, London, ON, Canada

^5^ Department of Human Genetics, Amsterdam Reproduction & Development Research Institute, Amsterdam University Medical Centers, University of Amsterdam, Amsterdam, The Netherlands

^6^ Institute of Medical Genetics, University of Zurich, 8952 Schlieren, Switzerland.

^7^ Université Paris Cité, Service de Médecine Génomique des Maladies Rares, INSERM UMR 1163, Institut Imagine, Hôpital Necker - Enfants Malades, Assistance Publique-Hôpitaux de Paris, Paris, France.

^8^ Normandy University, UNIROUEN, INSERM U1245 and University Hospital of Rouen, Department of Genetics and Reference Centre for Developmental Disorders, F 76000, Normandy Centre for Genomic and Personalized Medicine, Rouen, France

^9^ Laboratoire Cerba, Saint-Ouen-l'Aumône, France

^10^ Centre de Référence Anomalies Du Développement et Syndromes Malformatifs, FHU TRANSLAD, CHU Dijon, 21000, Dijon, France.

^11^ Center of Genetics and Reference Centre for Intellectual Disabilities, Dijon Bourgogne University Hospital, Dijon, France

^12^ Division of Medical Genetics, Department of Specialized Medicine, McGill University Health Centre, Montreal, Quebec, Canada

^13^ Department of Human Genetics, McGill University, Montreal, Quebec, Canada.

^14^ Laboratoire Multisites SeqOIA, Paris, France

^15^ Nantes Université, CHU de Nantes, CNRS, INSERM, l’institut du thorax, F-44000 Nantes, France

^16^ Nantes Université, CHU de Nantes, Service de Génétique médicale, F-44000 Nantes, France

^17^ Centre de Génétique Humaine, Institut de Pathologie et Génétique, Gosselies, Belgium.

^18^ Service de Génétique Médicale, Centre Labellisé Anomalies du Développement de l’Ouest, CHU de Rennes, Rennes, France

^19^ Montpellier University, Inserm U1183, Montpellier, France

^20^ Inserm UMR1231 GAD, Génétique des Anomalies du Développement, Université de Bourgogne, Dijon, France

^21^ Hunter Genetics, Waratah, NSW, Australia

^22^ Division of Medical Genetics, Nemours/A.I. DuPont Hospital for Children, Wilmington, DE, USA

^23^ Department of Genetics, Sart Tilman University Hospital, Belgium

^24^ UF de Génétique Clinique et Centre de Référence Maladies Rares des Anomalies du Développement et Syndromes Malformatifs, ERN ITHACA, APHP.Sorbonne Université, Hôpital Armand Trousseau, Paris, France

^25^ Department of Medical Genetics, Kaiser Oakland Medical Center, Oakland, California

^26^ APHP Sorbonne Université, Département de Génétique, Centre de Référence Déficiences Intellectuelles de Causes Rares, Paris, France

^27^ Department of Genetics, University Hospital of Toulouse, Toulouse, France

^28^ Department of Clinical Genetics, Leiden University Medical Center, Leiden, The Netherlands.

^29^ Blueprint Genetics, a Quest Diagnostics Company, 2505 3rd Ave, Suite 204, Seattle, 98121, USA

^30^ Greenwood Genetic Center, Greenwood, USA27 Division of Medical Genetics, Department of Specialized Medicine, McGill University Health Centre, Montreal, Quebec, Canada; Department of Human Genetics, McGill University, Montreal, Quebec, Canada.

**Supplemental Materials and Methods**

**DNA methylation analysis**

**Study cohort**

Blood-derived DNA samples were obtained from 25 individuals (14 males and 11 females). Fourteen samples were utilized for episignature discovery (discovery cohort). We used the discovery cohort for the purpose of probe selection and construction of the classification model for the episignature. All the individuals involved in the discovery cohort had confirmed pathogenic variants in *ARID2*. The remaining samples were utilized for episignature validation (n=11; validation cohort). The validation cohort included three samples with germline pathogenic variants in *ARID2*, two samples with a mosaic pathogenic variant, three with VUS variants in *ARID2* and three samples from previously undiagnosed patients in the EpiSign^TM^ Knowledge Database (EKD) (Table S2). Variants were classified according to the American College of Medical Genetics (ACMG) guidelines and Association for Molecular Pathology (AMP)^1,2^.

**DNA methylation data**

Bisulfite-converted genomic DNA from peripheral blood was analyzed using the Infinium Methylation EPIC Bead Chip array (San Diego, CA). The intensity data files (IDATS) containing methylated and unmethylated signal intensities were preprocessed and imported into R (version 4.2.3) ^3^ using the minfi R package (version 1.44.0). Standard preprocessing for Illumina microarrays was applied, including background correction, normalization, and quality control measures such as evaluating density plots and checking for sex and age discordance. Probes overlapping with single-nucleotide variations, cross-reactive probes, X or Y chromosome-specific probes, and probes with detection p-values >0.1 were filtered out, resulting in a final number of 772,557 probes.

**DNA methylation analyses**

DNA methylation analyses were conducted following established methods. ^4,5^ Matched controls were selected from the EKD based on age, sex, batch, and array type using the R package MatchIt.^6^ Samples with batch effects or >5% probe failure were excluded. Principal component analysis (PCA) was used to assess data structure and outliers in the training cohort and matched case-control samples. Feature selection was performed using matched cases and controls, followed by differential methylation analysis using linear regression fitting with the limma package. ^7^ Methylation beta values were used as predictors, adjusting for estimated blood cell counts as confounding variables. False discovery control was achieved using the empirical Bayes method and adjusted with the Benjamini-Hochberg procedure. Probe sets were varied based on top P-values, and variable importance was assessed using receiver operating characteristic curve analysis and correlation. Heatmaps and multidimensional scaling (MDS) were used to investigate separate clustering of cases and controls with the R package ggplots2. The best clustering was selected based on parameter values, and reproducibility was examined using leave-one-out cross-validation and unsupervised clustering results. Additionally, differentially methylated regions (DMRs) were identified using the R package DMRcate ^8^ with defined criteria of at least 5 CpGs within 1 kb distance and at least 0.1 absolute mean methylation difference between cases and controls. Results were further filtered using a Fisher P-value cutoff of 0.01.

**Prediction model**

The sensitivity and specificity of the *ARID2* episignature cohort were investigated with a classifier using all the episignature probes. We trained a support vector machine (SVM) model with the R package e1071 (version 1.7-13) using the selected features and the matched controls and cases as training data. To create more specificity, 75% of the samples (known to have an episignature, unaffected samples, and training controls) in the EKD were included, the other 25% were used as testing. We repeated these four times so that every sample was used as a testing sample one time. The average SVM, also named the methylation variant pathogenicity (MVP) score, was then used.

**Overlap of the ARID2 Genome-Wide DNA Methylation Profile with Other Neurodevelopmental Disorders on EpiSign™**

The functional annotation and EpiSignTM cohort comparison were performed based on previously published articles ^4^. Briefly, the percentage of DMPs shared between the ARID2 episignature and the other 56 neurodevelopmental disorder episignatures on the EpiSignTM clinical classifier were assessed and heatmaps and circos plots produced. Heatmaps were plotted using the R package pheatmap (version 1.0.12) and the circos plots were generated with the R package circlize (version 0.4.15)^9^. Clustering analysis was performed to search for relationships across all the 57 cohorts with known episignatures. Using the R package TreeAndLeaf (version 1.6.1) ^10^, we generated a tree and leaf plot to visualize the distance and similarities between the cohorts. To investigate the genomic location of the DMPs selected in the ARID2 cohort, we annotated probes in relation to CpG islands (CGIs) and genes with the R package annotatr (version 1.20.0) ^11^ with AnnotationHub (version 3.2.2) as described previously by Levy et al.^4^

**Exome sequencing**

***Individual 1:***

Libraries of genomic DNA samples were prepared using the Agilent Sureselect Human All Exon v5 kit (Agilent Technologies, Santa Clara, CA), and were sequenced on a HiSeq instrument (Illumina, San Diego, CA) according to the manufacturer’s recommendations for paired-end 76-bp reads. BAM files had been aligned to a human genome reference sequence (GRCh37/hg19) using BWA (Burrows–Wheeler Aligner; v0.7.15).

***Individual 2 and 11:***

The analyses were carried out within the DISSEQ research protocol. A panel of 459 genes involved in cognitive disorders was performed. Capture and sequencing were performed using the SureSelect QTX, Agilent and flow sequencing method (HiSeq 4000 sequencer, CHRGH platform). This analysis was followed by exome sequencing (Agilent_V5_51Mb, HiSeq 4000 sequencer), through the FHU TRANSLAD platform, with FastQC software. The sequences were aligned with the reference genome (GRCh37/Hg19) using BWA. Quality control was done using Picard and Genome analysis Toolkit software. Genetic variation affecting coding regions and splicing sites were identified using GATK. Structural and copy number (CNV) anomalies were identified using XHMM software.

***Individual 3:***

Trio whole exome sequencing was performed with Illumina HiSeq at BGI-Europe, after enrichment with the Agilent SureSelectXT Human All Exon 50Mb Kit. After read alignment (BWA) and variant calling with GATK (SNV) and CoNIFER (CNVs), annotation was done.

***Individual 4:***

Not available

***Individual 5:***

The exomic DNA was enriched by SureSelect Human All Exon V5 kit (Agilent) and subsequently analysed by the sequencing-by-synthesis technology (Illumina). Variants were detected with an in-house pipeline and annotated and filtered based on variants databases and HPO terms (MOON software, diploid).

***Individual 6:***

Genomic DNA was extracted from peripheral blood using CheMagic DNA purification kit. We used a sequencing panel targeting 503 genes associated to neurodevelopmental disorders. Paired-end sequence data sets from NextSeq500 (Illumina) runs were processed in 3 main steps: alignment against the human genome (hg19), variant calling for SNPs and small base insertions or deletions using SAMtools  and GATK, and variant annotation based on the Ensembl human database (GRCh37). Data were integrated in a  pipeline enabling copy number variant analysis based on double normalization of depth coverage. Variants were described according to HGVS variant nomenclature guidelines (http://varnomen.hgvs.org/) (den Dunnen et al., 2016), using the reference sequence RefSeq NM_152641.4. Sanger sequencing confirmed single nucleotide variants and segregation analysis were performed in each family.

***Individual 7:***

Trio WES was performed using the Roche MedExome prep kit, followed by 150 bases of paired- end reads via a massively parallel sequencing approach on Illumina NextSeq 500. Sequence reads were mapped to the human genome build (hg19 / GRCh37) by using the BWA tool. Validation of the candidate variant and segregation analysis in the family members were performed by Sanger sequencing.

***Individual 8 and 9:***

Diagnostic trio exome sequencing was performed on DNA extracted from peripheral blood using the xGen® Exome Research Panel v2.0 (IDT) for capturing followed by paired-end sequencing of 150 bp forward and 150 bp reverse using a NovaSeq 6000 S1 Reagent Kit (300 cycles) on a NovaSeq 6000 sequencer (Illumina Inc.). Raw fastQ files were aligned to the hg19 reference genome using the NextGene V2.4.2.3 software (SoftGenetics, State College, PA, USA). Coding regions and exon-intron boundaries up to 6 bp of all known disease-associated genes were analyzed. Coverage of at least 20fold was reached for 97.7% of base pairs. Variants were classified according to ACMG guidelines.

***Individual 10:***

WES was performed with the SureSelect Human All Exon V7 kit (Agilent, Santa Clara, CA, USA), followed by paired-end reads sequencing on an HiSeq1500 or a NetxSeq500 instrument (Illumina, San Diego, CA). Sequencing data have been analyzed according Thevenon *et al.* pipeline (Clin Genet. 2016;89:700-7). The variant has been confirmed by an alternative method as Sanger.

***Individual 12:***

Genome sequencing was performed at the SeqOIA laboratory (LBMS SeqOIA, Paris, France) using the same protocols for families C and D. FASTQ files were obtained from the bcl2fastq demultiplexing tool (v2.20.0.422, Illumina) and aligned to the GRCh38.92 genomic reference using BWA-MEM (v0.7.15). Haplotype Caller, GATK (v4.1.7.0) was used to call the SNV and delins (<50bp); variants were annotated by SNPEff (v4.3t). Further details are available on the website of the LBMS SeqOIA platform (<https://laboratoire-seqoia.fr/>).

***Individual 13:***

Trio WES was performed in a CAP and ISO accredited laboratory (Blueprint Genetics, Finland). The exonic and selected non-coding regions were captured with IDT xGen Exome Research Panel with custom-designed capture probes, followed by paired-end sequencing (150 by 150 bases) using the Illumina sequencing system (NovaSeq). Clean sequence reads were mapped to the human reference genome (GRCh37/hg19).

***Individual 14:***

Diagnostic single exome sequencing was performed on DNA extracted from peripheral blood using the Agilent SureSelectXT Kit (V6) for capturing followed by paired-end sequencing of 125 bp forward and 125 bp reverse using a HiSeq SBS Kit v4 on a HiSeq2500 sequencer (Illumina Inc.). Raw fastQ files were aligned to the hg19 reference genome using the NextGene V2.4.1.1 software (SoftGenetics, State College, PA, USA). Coding regions and exon-intron boundaries up to 6 bp of all known genes associated with Noonan syndrome, developmental delay, short stature, or congenital heart defect were analyzed. Coverage of at least 20 fold was reached for 94.9% of base pairs. Sanger sequencing was used to confirm the detected ARID2 variant in the patient and for segregation studies in both parents.

***Individual 15:***

WES was performed with *Twist Human Core Exome Plus Kit (ref 100803 + 101022), Twist BioSciences,* followed by *sequencing: 101bp read on a NextSeq550 sequencer (Illumina®) (Gnirke, Nat Biotechnol 2009. 27, 182-189) using the NextSeq™ 500/550 High Output v2 kit.*,. Sequence reads were mapped to *human reference genome (GRCh38/hg38). The sequencing depth of coding exons and splice sites (source: RefSeq) was calculated using as quality thresholds a percentage of bases covered by at least 20 reads greater than 98% and an average read depth greater than 50x. Bioinformatic analysis: Local realignment of insertions and deletions: GATK-IndelRealigne. Identification of SNV and InDels germline genetic alterations: GATK Haplotype Caller GVCF (Broad Institute). Technical quality control: quality of alignments, bases read, elimination of duplicate reads, measurement of coverage rate, homogeneity, and depth read per sample (using FastQC, Picard Tools, and GATK base quality score recalibration and IntegraGen-specific tools). Selection of variants located in the coordinates of the capture kit's target exons +/-25 bases. Omics" data analysis and functional annotation of variants using Variant Effect Predictor (VEP) provided by Ensembl. Interpretation and prioritization of data in the Sirius interface (Integragen).*

***Individual 16:***

WES was performed with the SureSelect Clinical Research Exome kit (Agilent, Santa Clara, CA, USA), followed by 75-bp paired-end reads on an Illumina HiSeq2000. Sequence reads were mapped to the huan genome build (hg19 / GRCh37) byusing the BWA tool. (v0.7.3).

***Individual 17:***

WES was performed with the SureSelect Clinical Research Exome kit (Agilent, Santa Clara, CA, USA), followed by 75-bp paired-end reads on an Illumina HiSeq2000. Sequence reads were mapped to the human genome build (hg19 / GRCh37) byusing the BWA tool. (v0.7.3).

***Individual 18:***

Exome capture and sequencing were performed at Integragen SA from 1 µg of genomic DNA per individual using the TWIG kit on a NovaSeq 6000 (Illumina) according to manufacturer’s instructions. 75-bp paired-end reads were generated, that were aligned to the human genome reference sequence (GRCh37/hg19 assembly) using Burrows-Wheeler aligner (BWA; version 0.7.15 or 0.7.3). Duplicate reads were marked using Picard MarkDuplicates (version 2.4.1) (http://broadinstitute.github.io/picard/) and aligned read were then processed using GATK BaseRecalibrator and PrintReads (Genome Analysis Toolkit; version 3.8 or 3.4) to recalibrate base quality scores, according to GATK Best Practices recommendations. Quality control was performed on all BAM files by calculating depth of coverage onto RefSeq database (release 2018-11-11) with GATK DepthOfCoverage. SNPs and indels were identified from BAM files using GATK HaplotypeCaller. All variants identified were annotated using SnpEff (version 4.3).

***Individual 19:***

Genome sequencing was performed at the SeqOIA laboratory (LBMS SeqOIA, Paris, France) using the same protocols for families C and D. FASTQ files were obtained from the bcl2fastq demultiplexing tool (v2.20.0.422, Illumina) and aligned to the GRCh38.92 genomic reference using BWA-MEM (v0.7.15). Haplotype Caller, GATK (v4.1.7.0) was used to call the SNV and delins (<50bp); variants were annotated by SNPEff (v4.3t). Further details are available on the website of the LBMS SeqOIA platform (https://laboratoire-seqoia.fr/).

***Individual 20:***

Trio whole exome sequencing (WES) was performed at Integragen (Evry, France). Whole- exome capture was performed using an Agilent in-solution enrichment methodology (SureSelect Clinical Research Exome V2, Agilent Technologies), followed by 75 bases of paired- end reads via a massively parallel sequencing approach on Illumina HiSeq4000. Sequence reads were mapped to the human genome build (hg38 / GRCh38) by using the Burrows- Wheeler Aligner (BWA) tool. Validation of the candidate variant and segregation analysis in the family members were performed by Sanger sequencing.

***Individual 21:***

Exome Sequencing: Libraries of genomic DNA samples were prepared using the Twist Human Core Exome kit (Twist Biosciences, San Francisco, CA), and were sequenced on a NovaSeq 6000 instrument (Illumina, San Diego, CA) according to the manufacturer’s recommendations for paired-end 151-bp reads. A mean depth of 101.72x and 88.68 was reach for dijex5745 and dijex6180. 96.6 % (dijex5745) and 97.1 % (dijex6180) of the refseq exons were covered at least by 10 reads.

Bioinformatics: Variants were identified using a computational platform of the FHU Translad, hosted by the University of Burgundy Computing Cluster (CCuB). Raw data quality was evaluated by FastQC software (v0.11.4). Reads were aligned to the GRCh37/hg19 human genome reference sequence using the Burrows-Wheeler Aligner (v0.7.15). Aligned read data underwent the following steps: (a) duplicate paired-end reads were removed by Picard software (v2.4.1), and (b) base quality score recalibration was done by the Genome Analysis Toolkit (GATK v3.8) Base recalibrator. Using GATK Haplotype Caller, Single Nucleotide Variants with a quality score >30 and an alignment quality score >20 were annotated with SNPEff (v4.3). Rare variants were identified by focusing on nonsynonymous changes present at a frequency less than 1% in the GNOMAD database. Copy Number Variants were detected using xHMM (v1.0) and were annotated using in-house python scripts. They were filtered regarding their frequency in public databases (DGV, ISCA, DDD).

***Individual 22:***

Search for genomic imbalance by Shallow Whole Genome Sequencing (SWGS*) from DNA (BIOOS- Illumina NextSeq-Novaseq kits). This analysis allows the detection and analysis of genomic variations such as CNV (Copy Number Variation).

***Individual 23:***

WES was performed at GeneDx Laboratories, using Next Generation sequencing/Illumina Hi Seq. Variant detection using a custom analysis tool (Xome Analyzer).

***Individual 24:***

Not available

**Array-CGH**

***Individual 25:***

An Array-CGH was performed using a 4x180K Agilent oligonucleotide micro-array with a theoretical resolution of 70 kb (5 consecutive probes), computer analysis performed by Agilent Feature Extraction and Cytogenomics software, and same-sex reference DNA (GRCh37). The deletion was confirmed in the patient and research in the parents by hybridization in situ using CTD-2338L16 (12q12)/CTD-3011H14(12p13.32) probes.

***Individual 26:***

An Array Comparative Genome Hybridization (Array-CGH) was performed using a CYTOSCAN HD (SNP AFFYMETRIX) micro-array with a theoretical resolution of 200 kb. Analysis was performed by Cyto-B-N2.0.1.2 (r5919) NetAfx Build 33.1 (hg19/GRCh37). The presence of this deletion was confirmed and sought in his both parents by FISH analysis using the BAC RP11- 19E18.

***Individual 27:***

After extraction of genomic DNA from a peripheral blood sample, high-resolution chromosomal microarray analysis was performed on the patient and the mother using an Affymetrix Cytoscan 2.7 M Array (annotation v. 30-spx) (Affymetrix, Santa Clara, CA, USA). The analysis was performed using Affymetrix Chromosome Analysis Suite 3.1 in comparison to 820 healthy controls at a resolution of 50 kb for duplications and approx. 5-10 kb for deletions.

**VUS**

***Individual 29***

No available

***Individual 30:***

Not available

***Individual 31:***

Sequencing libraries were prepared by ligating sequencing adapters to both ends of DNA fragments and were size-selected with bead-based method to ensure optimal template size and amplified by polymerase chain reaction (PCR). Regions of interest (exons and intronic targets) were targeted using hybridization-based target capture method. Sequencing libraries were sequenced using the Illumina's sequencing-by-synthesis method using paired-end sequencing (150 by 150 bases). Primary data analysis converting images into base calls and associated quality scores was carried out by the sequencing instrument using Illumina's proprietary software, generating CBCL files as the final output. Sequence reads of each sample were mapped to the human reference genome (GRCh37/hg19). Burrows-Wheeler Aligner (BWA-MEM) software was used for read alignment. Duplicate read marking, local realignment around indels, base quality score recalibration and variant calling were performed using GATK algorithms (Sentieon) for nDNA. Variant data for was annotated using a collection of tools (VcfAnno and VEP) with a variety of public variant databases including but not limited to gnomAD, ClinVar and HGMD. The variant classification follows the Blueprint Genetics Variant Classification Schemes modified from the ACMG guideline 2015. For missense variants, in silico variant prediction tools such as SIFT, PolyPhen, MutationTaster were used to assist with variant classification.

**Supplemental figures**


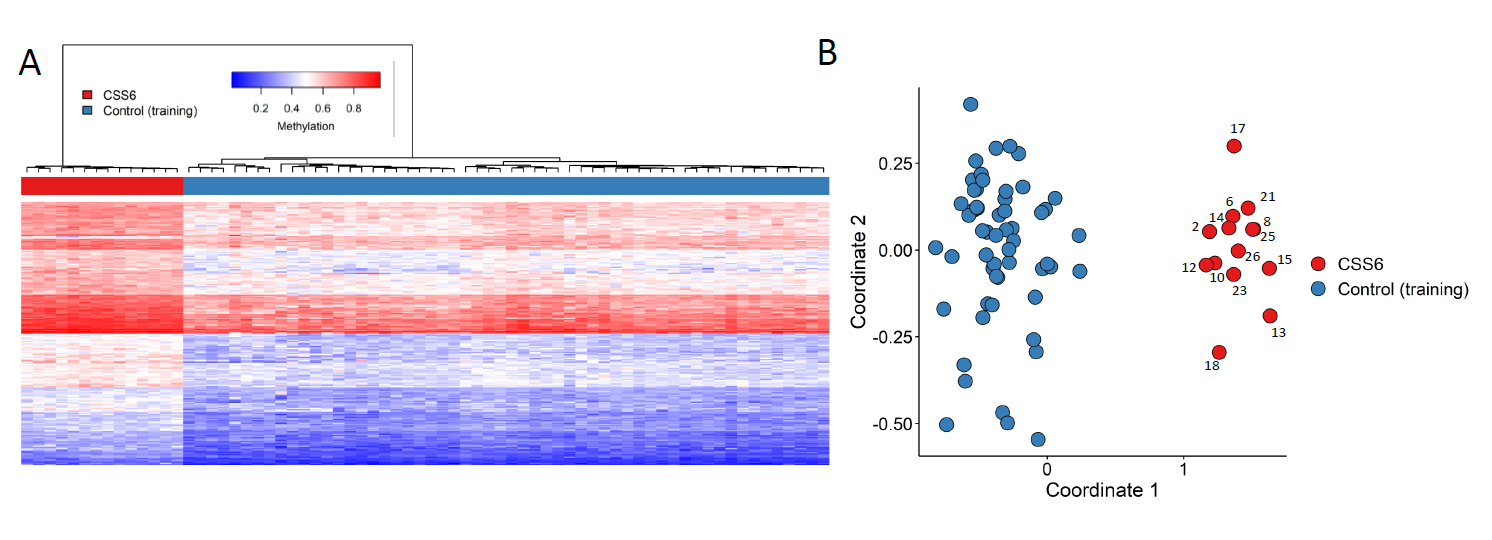


**Figure S1: Coffin-Siris syndrome-6 (ARID2) episignature discovery cohort.** (A) Euclidean hierarchical clustering heatmap, each column represents one ARID2 discovery case or control, each row represents one probe selected for this episignature. It shows a clear separation between the cases in red and controls in blue. (B) Multidimensional scaling (MDS) plot shows segregation of ARID2 cases and controls.


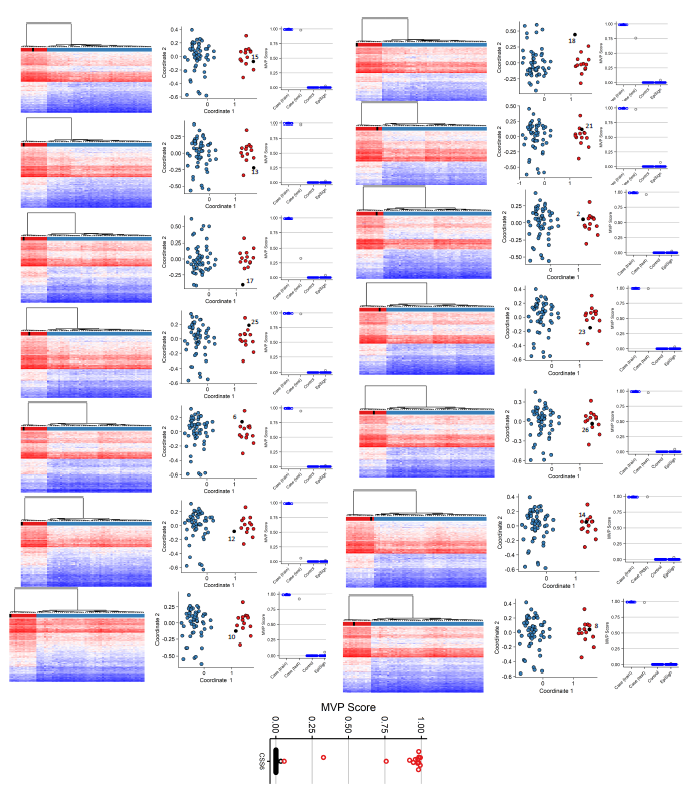


**Figure S2: Leave-one-out cross validation.** In each cross-validation set, a single test case sample (dark blue) is used as testing. The other ARID2 cases used for episignature training are shown in red and control training sample shown in blue in the heatmap and MDS plots. The last plots demonstrate the MVP scores of the Support Vector Machine (SVM) classifier model that was trained using the selected ARID2 episignature probes from training cases, 75% of controls and other EpiSign samples (blue). The remaining 25% of controls and other disorder samples were used as testing alongside the ARID2-test case (grey).


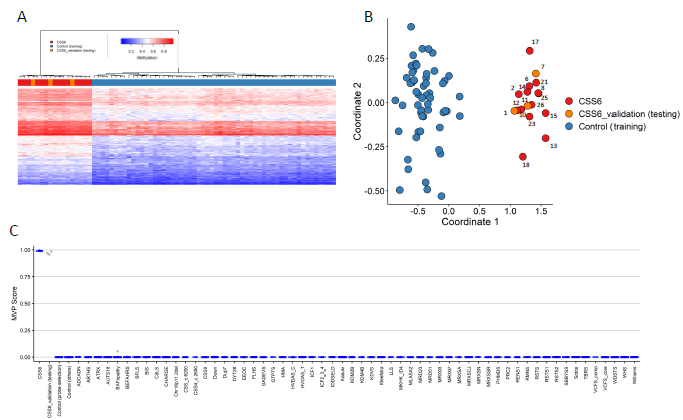


**Figure S3: Coffin-Siris syndrome-6 (ARID2) episignature discovery + validation cohort.** (A) Euclidean hierarchical clustering heatmap, each column represents one ARID2 discovery/ validation case or control, each row represents one probe selected for this episignature. This heatmap shows segregation of the ARID2 validation cases (orange) with the ARID2 training (discovery) cases (red) from controls (blue). (B) Multidimensional scaling (MDS) plot shows segregation of ARID2 cases (validation and discovery) from controls. (C) Support Vector Machine (SVM) classifier model. The model was trained using the selected episignature probes, 75% of controls and 75% of other neurodevelopmental disorder samples (blue). The remaining 25% controls and 25% of other disorder samples were used for testing (grey). Plot shows the ARID2 discovery cases with a methylation variant pathogenicity (MVP) score close to 1 compared with all other samples, showing the specificity of the classifier and episignature.


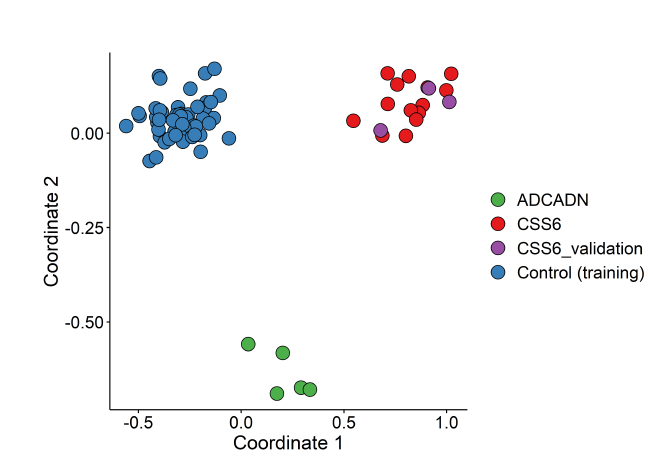


**Figure S4: Overlap between the ADCADN episignature and the CSS6 episignature**


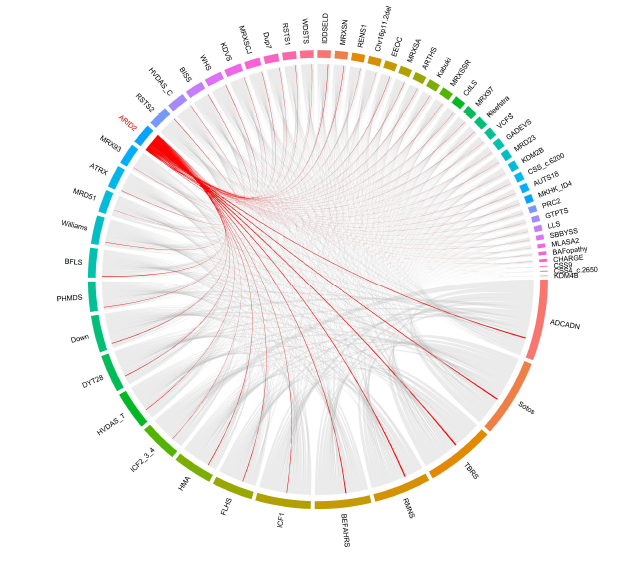


**Figure S5: Differentially methylated probes (DMPs) shared between the ARID2 cohort and 56 other episignatures on EpiSign™.** Circos plot representing the probes shared between each pair of cohorts. The thickness of the connecting lines indicates the number of probes shared between the two cohorts. ARID2 connections are highlighted in red.

**References**

1. Richards, S. *et al.* Standards and guidelines for the interpretation of sequence variants: a joint consensus recommendation of the American College of Medical Genetics and Genomics and the Association for Molecular Pathology. *Genet. Med. Off. J. Am. Coll. Med. Genet.* **17**, 405–424 (2015).

2. Riggs, E. R. *et al.* Technical standards for the interpretation and reporting of constitutional copy number variants: a joint consensus recommendation of the American College of Medical Genetics and Genomics (ACMG) and the Clinical Genome Resource (ClinGen). *Genet. Med. Off. J. Am. Coll. Med. Genet.* **22**, 245–257 (2020).

3. Aryee, M. J. *et al.* Minfi: a flexible and comprehensive Bioconductor package for the analysis of Infinium DNA methylation microarrays. *Bioinforma. Oxf. Engl.* **30**, 1363–1369 (2014).

4. Levy, M. A. *et al.* Novel diagnostic DNA methylation episignatures expand and refine the epigenetic landscapes of Mendelian disorders. *HGG Adv.* **3**, 100075 (2022).

5. Aref-Eshghi, E. *et al.* Diagnostic Utility of Genome-wide DNA Methylation Testing in Genetically Unsolved Individuals with Suspected Hereditary Conditions. *Am. J. Hum. Genet.* **104**, 685–700 (2019).

6. Ho, D., Imai, K., King, G. & Stuart, E. A. MatchIt: Nonparametric Preprocessing for Parametric Causal Inference. *J. Stat. Softw.* **42**, 1–28 (2011).

7. Ritchie, M. E. *et al.* limma powers differential expression analyses for RNA-sequencing and microarray studies. *Nucleic Acids Res.* **43**, e47 (2015).

8. Peters, T. J. *et al.* De novo identification of differentially methylated regions in the human genome. *Epigenetics Chromatin* **8**, 6 (2015).

9. Gu, Z., Gu, L., Eils, R., Schlesner, M. & Brors, B. circlize Implements and enhances circular visualization in R. *Bioinforma. Oxf. Engl.* **30**, 2811–2812 (2014).

10. Cardoso, M. A. *et al.* TreeAndLeaf: an R/Bioconductor package for graphs and trees with focus on the leaves. *Bioinforma. Oxf. Engl.* **38**, 1463–1464 (2022).

11. Cavalcante, R. G. & Sartor, M. A. annotatr: genomic regions in context. *Bioinforma. Oxf. Engl.* **33**, 2381–2383 (2017).
